# Microbial Forensics: Predicting Phenotypic Characteristics and Environmental Conditions from Large-Scale Gene Expression Profiles

**DOI:** 10.1371/journal.pcbi.1004127

**Published:** 2015-03-16

**Authors:** Minseung Kim, Violeta Zorraquino, Ilias Tagkopoulos

**Affiliations:** 1 Department of Computer Science, University of California, Davis, Davis, California, United States of America; 2 UC Davis Genome Center, University of California, Davis, Davis, California, United States of America; Princeton University, United States of America

## Abstract

A tantalizing question in cellular physiology is whether the cellular state and environmental conditions can be inferred by the expression signature of an organism. To investigate this relationship, we created an extensive normalized gene expression compendium for the bacterium *Escherichia coli* that was further enriched with meta-information through an iterative learning procedure. We then constructed an ensemble method to predict environmental and cellular state, including strain, growth phase, medium, oxygen level, antibiotic and carbon source presence. Results show that gene expression is an excellent predictor of environmental structure, with multi-class ensemble models achieving balanced accuracy between 70.0% (±3.5%) to 98.3% (±2.3%) for the various characteristics. Interestingly, this performance can be significantly boosted when environmental and strain characteristics are simultaneously considered, as a composite classifier that captures the inter-dependencies of three characteristics (medium, phase and strain) achieved 10.6% (±1.0%) higher performance than any individual models. Contrary to expectations, only 59% of the top informative genes were also identified as differentially expressed under the respective conditions. Functional analysis of the respective genetic signatures implicates a wide spectrum of Gene Ontology terms and KEGG pathways with condition-specific information content, including iron transport, transferases, and enterobactin synthesis. Further experimental phenotypic-to-genotypic mapping that we conducted for knock-out mutants argues for the information content of top-ranked genes. This work demonstrates the degree at which genome-scale transcriptional information can be predictive of latent, heterogeneous and seemingly disparate phenotypic and environmental characteristics, with far-reaching applications.

## Author Summary

The transcriptional profile of an organism contains clues about the environmental context in which it has evolved and currently lives, its behavior and cellular state. It is yet unclear, however, how much information can be efficiently extracted and how it can be used to classify new samples with respect to their environmental and genetic characteristics. Here, we have constructed an extensive transcriptome compendium of *Escherichia coli* that we have further enriched via an iterative learning approach. We then apply an ensemble of various machine learning algorithms to infer environmental and cellular information such as strain, growth phase, medium, oxygen level, antibiotic and carbon source. Functional analysis of the most informative genes provides mechanistic insights and palpable hypotheses regarding their role in each environmental or genetic context. Our work argues that genome-scale gene expression can be a multi-purpose marker for identifying latent, heterogeneous cellular and environmental states and that optimal classification can be achieved with a feature set of a couple hundred genes that might not necessarily have the most pronounced differential expression in the respective conditions.

## Introduction

Genome-scale transcriptional profiling has become a standard and relatively inexpensive way to identify the overall cellular state and condition-specific cellular responses to external stimuli. For instance, different sets of genes are known to be active in each growth phase and medium [1], while strain polymorphisms can result in a remarkably diverse transcriptional repertoire [2,3]. Similarly, it is known that bacterial organisms undergoing rapid adaptations to varying environments, such as heat-shock and osmotic stress, produce differential expression profiles that are indicative of the corresponding stress [4–9]. Genome-wide transcriptional profiling can be thought of as a complex representation of all cellular functions and states, with a wealth of multiplexed information that, if decoded efficiently, can provide a fast and quite accurate all-encompassing snapshot of the cell and its environment.

Despite its obvious correlation with various physiological and cellular states, we lack a clear understanding of the information content related to the manifold phenotypes that can be extracted from the genome-scale transcription profiles. Until now, a significant obstacle was the absence of sufficient transcriptional data to support the training of multi-feature and multi-label classifiers. Indeed, after aggregating all high-throughput transcriptional data that is currently available for *E*. *coli*, the most well-studied model microbe, we are still limited to a few thousands microarray or RNA-Seq experiments that cover more than 30 strains, a dozen different media and a multitude of other genetic (knock-out, over-expressions, re-wirings), or environmental (carbon limitation, chemicals, abiotic factors) perturbations. Although this collection has already increased by an order of magnitude from the roughly two hundred genome-wide transcriptional profiles that we had eight years ago, it is still an inadequate sampling of the relevant experimental space. In addition, since these experiments have been performed in different technological platforms (e.g. Affymetrix *E*. *coli* Genome 2.0, Affymetrix *E*. *coli* Antisense) and technologies (e.g. microarrays vs. RNA-Seq), in different labs and under different environmental conditions, appropriate normalization schemes are both of paramount importance and with an added complexity. As such, efficient training of machine learning methods is hindered due to data complexity, compatibility and the curse of dimensionality that plagues datasets with thousands of features (genes) but only a few samples (conditions).

The application of high-dimensional prediction algorithms has been widespread in biology ranging from gene function prediction [10–12], disease risk estimation from inherited variants [13], and network inference [14–18], but the vast majority of these studies are confined to the use of transcriptional data on pathological, pharmacological and clinical predictions [19–25]. Interestingly, a *Saccharomyces cerevisiae* study that involved tens of data samples was able to predict growth rates [26], while a multi-class stressor prediction in rice used five hundred transcription profiles [27]. More recently, a probabilistic human tissue and cell type predictor was built based solely on gene expression profiles [28].

**Fig 1:**
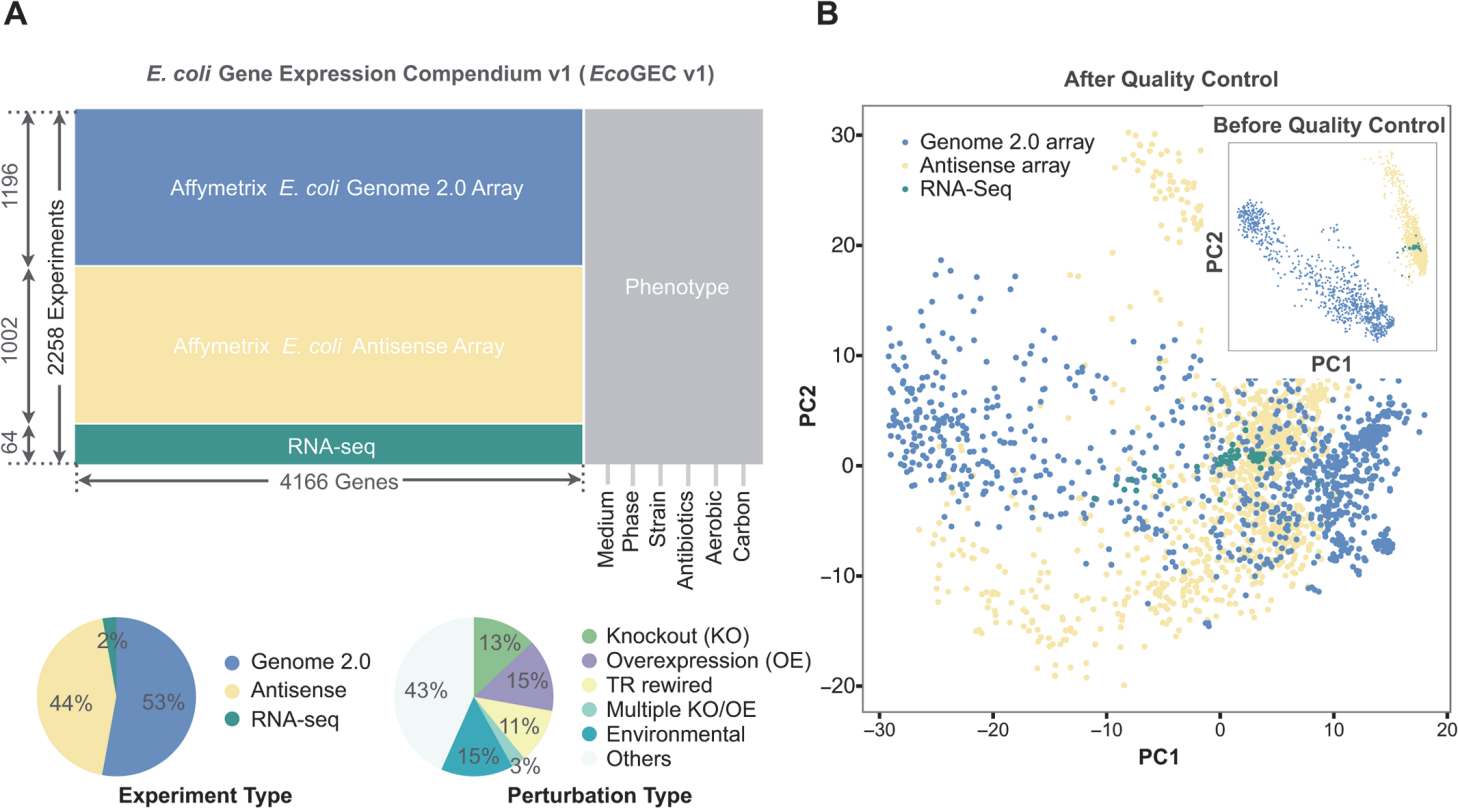
Compendium analysis and normalization. **(A)** The *E*. *coli* Gene Expression Compendium (*Eco*GEC) is constructed from raw genome-wise transcriptional data **(B)** Principal Component Analysis on the *Eco*GEC before (inset) and after (main) normalization through linear transformation. p1 and p2 represent first and second principal component respectively. Platform biases are corrected by performing platform-specific categorization of gene expression values.

**Fig 2:**
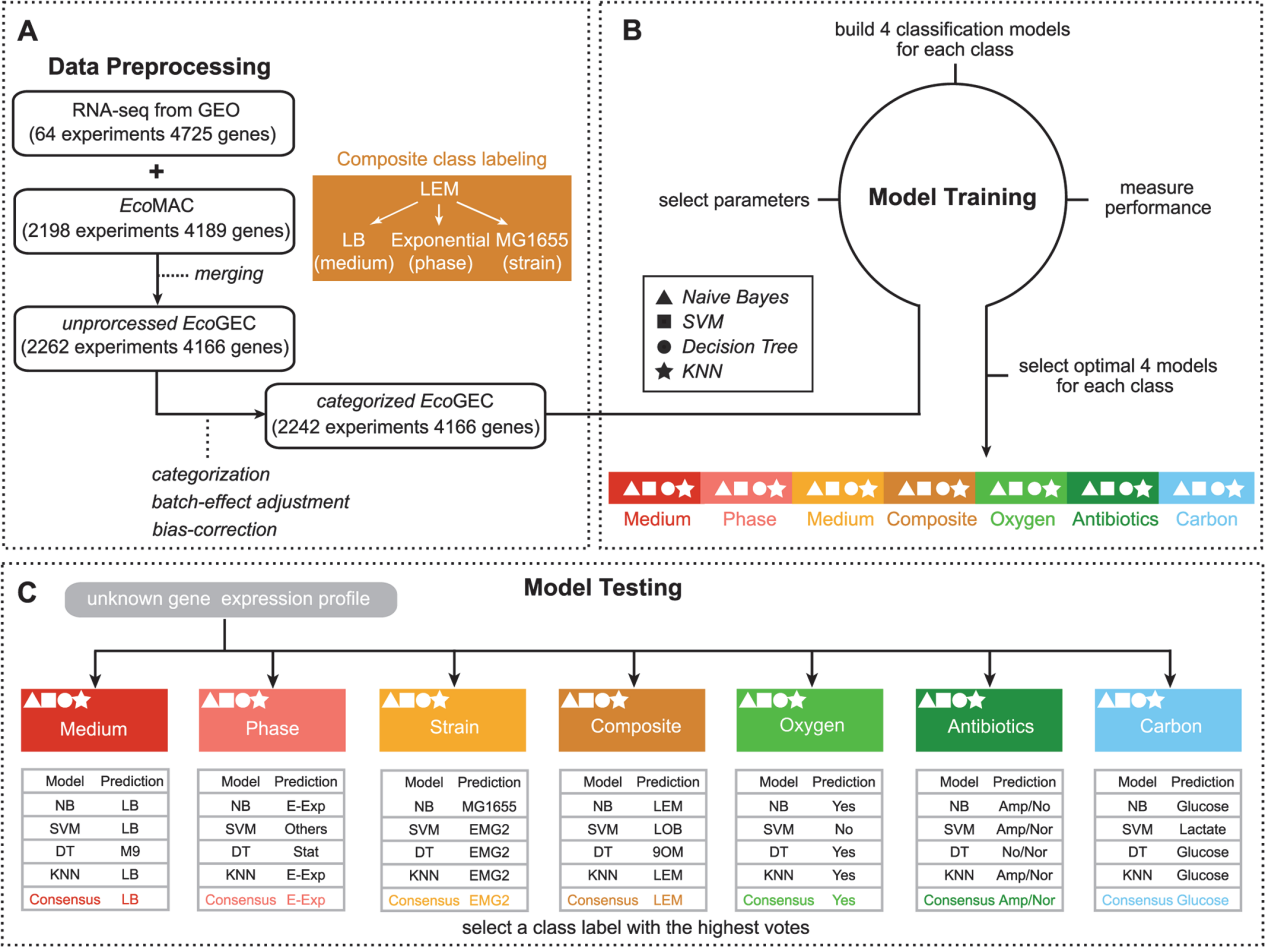
Gene expression compendium and classification workflow. The workflow is divided into three steps: **(A)** data preprocessing that combines RNA-Seq and microarray datasets. *EcoGEC* is categorized into three differential expression bins (under-expressed, UE; wild-type, WT; over-expressed OE) and pre-processed for batch-effect and bias correction. **(B)** model training, where parameters are trained based on four different machine learning methods for each of the classification tasks, and **(C)** model testing where new samples are assigned to the class labels that have the majority of votes from 4 prediction methods for each of the eight characteristic predictors.

In this work, we investigate how well we can predict cellular and environmental state from genome-wide expression, using known gene expression profiles as our only training data. We report the optimal number of features for each classification task, what these features are, and all relevant pathways. To achieve this, we have extended, normalized and annotated a compendium that was compiled recently [29] to incorporate all published high-quality Affymetrix microarray and RNA-Seq datasets in *E*. *coli* (2258 samples in total, Fig. 1A). This *E*. *coli* Gene Expression Compendium (*Eco*GEC), consists of publicly available data that were curated from online public databases such as GEO [30], ArrayExpress [31], SRA [32], SMD [33], M3D [34] and PortEco [35]. To increase the compatibility among the various arrays, we adjusted batch-effects across data from different sources and devised a statistical normalization scheme that significantly removed biases (see Methods; Fig. 1B, Table 1). Concomitantly, we developed an iterative learning procedure to impute unannotated or mis-labeled data and used it to increase the quality of the resulting datasets (Fig. 2A). By applying four different machine-learning algorithms on the *Eco*GEC compendium (Fig. 2B), we predicted six different organism and environmental variables from gene expression profiles related to medium, growth phase, strain, aerobic conditions, antibiotics and carbon sources present (Fig. 2C). Functional, network and mechanistic analysis of the highly-informative features provide a comprehensive map of the implicated genes and pathways.

**Table 1:** Class label distributions.

	Classes													
	Medium		Strain		Phase		Oxygen		Nor		Amp		Carbon	
**Class Labels**	LB	1356	MG1655	1368	E-Exp	148	Y	2178	Y	227	Y	56	Glucose	471
	M9	301	BW25113	148	ML-Exp	1368	N	64	N	2015	N	2186	Glycerol	94
	MOPS	86	EMG2	132	Stat	132							Acetate	49
	Others	499	Others	594	Missing	601							Others	1628
**Baseline**	60.4%		61%		61%		97.1%		89.8%		97.5%		72%	

## Results

### Accurate prediction of genetic and environmental parameters requires a small, informative gene set

**Fig 3:**
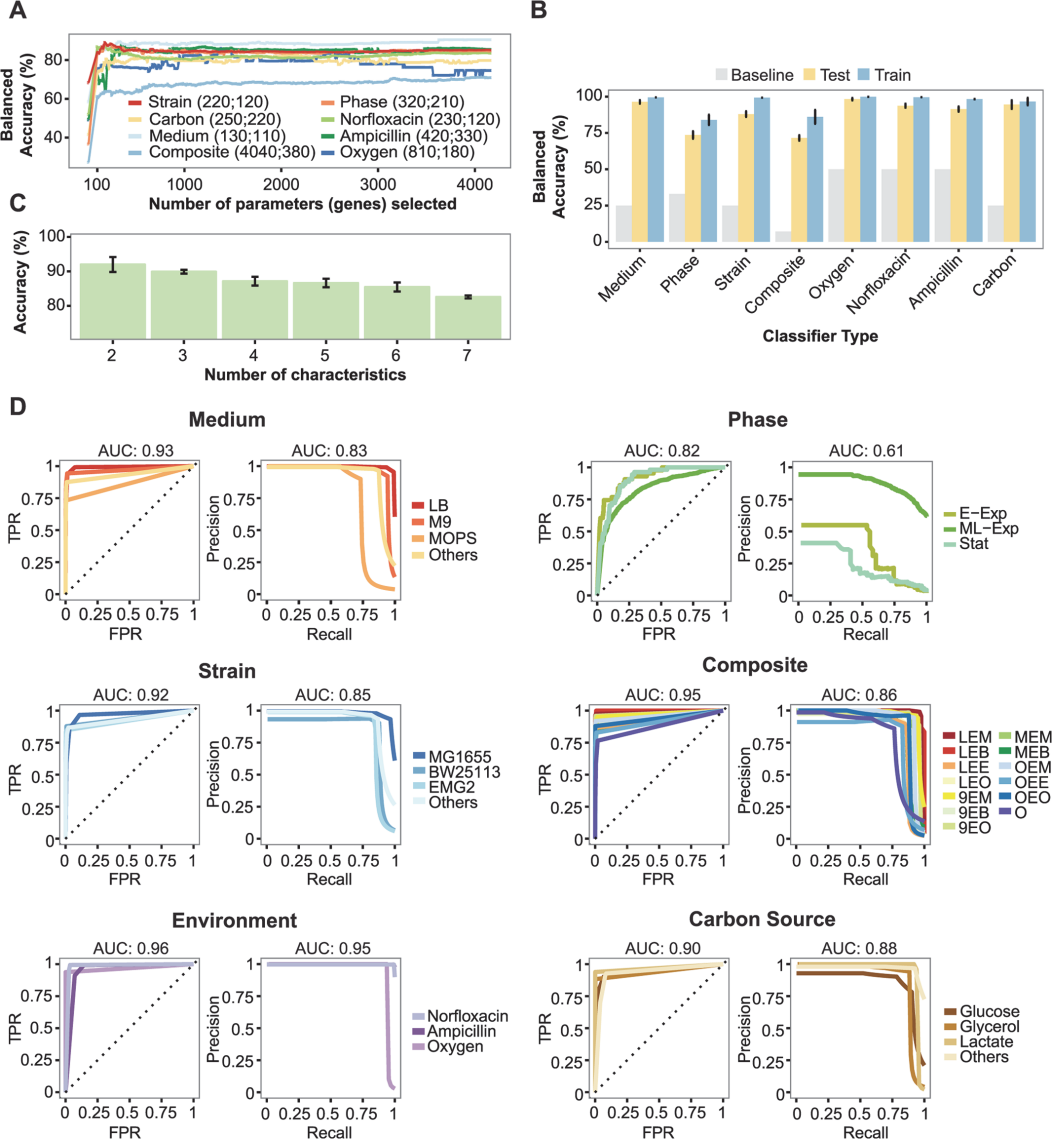
Classification performance. **(A)** Balanced accuracy in testing set for the 8 classification tasks as a function of number of genes selected. Genes (x-axis) are ordered by the mutual information of their expression to the predictor variable. For each classifier, the optimal number of features (derived from the training data) and the minimum number of genes at near-optimal (within 2%) classification are shown in the legend (first and second value, respectively). **(B)** Leave-one-batch-out cross-validation, with the training and testing balanced accuracy for each classifier is compared with the baseline. The baseline is estimated by dividing the maximum accuracy (100) by the number of classes for any given characteristic. **(C)** Combined multi-modal predictions using a set of individual classifiers. The parameter k represents the number of characteristics to be classified (two antibiotics, *aerobic or anaerobic respiration, medium, phase* and *strain*), represents all possible combinations and increases from 2 to 7 (x-axis). The average accuracy for each combination of k characteristics to be predicted is reported. **(D)** ROC curve (left) and PR curve (right) for predictor of each characteristic (TPR; true-positive rate, FPR; false-positive rate, E-Exp; early exponential phase, M/L-Exp; mid/late exponential phase, Stat; stationary phase).

We first investigated how many genes are required to achieve optimal performance and the minimum number of genes with near-optimal performance, defined as 2% reduction from the optimal balanced accuracy. As shown in Fig. 3A, in most cases the cumulative information content is asymptotically approaching a maximal value within a few hundred genes. The balanced accuracy profile of the different predictors spans a large spectrum of behaviors, from profiles that are optimal early on, such as in the case of the *medium* classifier where the 150 first genes are sufficient for accurate classification, to profiles that rise slowly, as in the case of the *composite* classifier, which is defined as the model that classify classifies 3 characteristics of medium, phase, strain altogether. In general, however, our results show that the subset of genes that is needed to achieve high balanced classification accuracy is neither a handful of biomarkers, nor a large gene set, with all cases achieving near-optimal performance with 100 to 400 genes. In the most extreme case of the composite classifier, a near-optimal balanced accuracy (70.26%) can be achieved with less than 400 genes, which is close to its maximum performance (71.55%) that is achieved when considering all 4166 genes. To investigate the relationship of data size with classification performance, we systematically reduced the dataset, keeping a balanced class/label distribution. Our results argue that although there is an expected reduction in classification performance, as the dataset is progressively reduced by up to 75%, the method is quite robust with an average reduction of 6% classification performance per quartile reduction in data size (S1 Table).

In all cases, the classification performance is significantly higher than the balanced baseline (Mann-Whitney-Wilkoxon test, *P* < 2.398 × 10^−3^), with the balanced accuracy of all classifiers ranging between 69.95% (±3.52%) to 98.27% (±2.32%) (Fig. 3B, S2 Table). For predicting the growth phase, we first imputed any unannotated phase information, which accounted for 34% of the compendium. We used a learning approach in which missing data is inferred iteratively. This preprocessing step was found to substantially increase the classification performance when evaluated across all classification tasks by an average of 7.3% and as much as 22% in some cases (S1 Fig., S2A Fig., S2C Fig., S3 Table, S4 Table). Interestingly, by following this approach, we were able to infer the characteristics from 90.6% of the unannotated phase data (S2B Fig.). The iterative learning method does not significantly decrease the MI levels that are observed when compared to those obtained from the original dataset and the gene ranking is mostly preserved (S5 Table, *Kendall tau rank* correlation: τ = 0.714, *P* < 2.2 10^−16^). The simultaneous prediction of all seven characteristics of a sample using seven individual classifiers yields an accuracy of 84.21% (±1.39%) (Fig. 3C). To create the necessary training set for the simultaneous prediction of three characteristics (*medium, phase* and *strain*), we had to reduce the amount of classes to 13 due to insufficient data (see Methods). Interestingly, the composite classifier that simultaneously selects one of the 13 classes, has an increased accuracy (71.55% ± 3.07%) to that of individual classifiers on the same class types (61.23% ± 2.33%) and it is significantly higher than the baseline (37% and 7.14% for balanced and imbalanced baseline accuracy, respectively). Altogether, the results suggest that multiple environmental and cellular features of an organism can be precisely predicted from a set of individual classifiers, by using a small, targeted gene set.

Table 2 and S6 Table contain the contingency tables of each classifier and Fig. 3D depicts the corresponding ROC and PR curves [36]. The overall AUC of the ROC curves exceeds 0.82, except in the case of stationary phase (0.71). This result is likely due to the high noise level and low sampling size for that class, which dilutes discriminatory features between the mid/late exponential and stationary phases. In the contingency table of the composite classifier (Table 2), the lowest classification case was observed in the case of “Others” (58/179 samples). This is expected, since that class corresponds to samples that either are missing data or represent classes that have low sample sizes and are grouped together.

**Table 2:** Contingency table of composite classifier.

		Predicted Medium/Phase/Strain													
		LEM	LEB	LEE	LEO	9EM	9EB	9EO	MEM	MEB	OEM	OEE	OEO	O	Total
**Known Medium/Phase/Strain**	**LEM**	822	0	0	1	0	0	0	0	0	0	0	0	8	831
	**LEB**	3	97	0	1	0	0	0	0	0	1	0	1	1	104
	**LEE**	2	0	41	0	0	0	0	0	0	0	0	0	11	54
	**LEO**	13	2	0	250	0	0	0	0	0	1	0	3	2	271
	**9EM**	0	0	0	1	215	0	0	0	0	0	0	0	0	216
	**9EB**	0	0	0	0	0	30	1	0	0	0	0	0	0	31
	**9EO**	0	0	0	0	1	0	49	1	0	0	0	0	1	52
	**MEM**	3	0	0	2	0	0	0	50	3	0	0	0	0	58
	**MEB**	0	0	0	1	0	0	0	1	22	0	0	0	0	24
	**OEM**	2	1	0	1	1	0	0	0	0	174	0	1	8	188
	**OEE**	0	0	1	0	0	0	0	0	0	0	29	0	5	35
	**OEO**	4	2	0	2	0	0	0	0	0	3	0	186	2	199
	**O**	31	9	15	18	0	0	2	1	0	20	3	22	58	179
	**Total**	880	111	57	277	217	30	52	53	25	199	32	213	96	2242

(1) LEM, LB medium + mid/late exponential phase + MG1655; (2) LEB, LB medium + mid/late exponential phase + BW25113; (3) LEE, LB medium + mid/late exponential phase + EMG2; (4) LEO, LB medium + mid/late exponential phase + strains other than MG1655, BW25133 and EMG2; (5) 9EM, M9 + mid/late exponential phase + MG1655; (6) 9EB, M9 + mid/late exponential phase + BW25113; (7) 9EO, M9 + mid/late exponential phase + strains other than MG1655, BW25133 and EMG2; (8) MEM, MOPS + mid/late exponential phase + MG1655; (9) MEB, MOPS + mid/late exponential phase + BW25113; (10) OEM, the other medium that is not LB, M9, or MOPS + mid/late exponential phase + MG1655; (11) OEE, the other medium that is not LB, M9, and MOPS + mid/late exponential + EMG2; (12) OEO, the other medium that is not LB, M9, or MOPS + mid/late exponential phase + the other strain that is not MG1655, BW25113, or EMG2; (12) O, the others that don’t belong to any of thirteen classes

### Biomarker discovery through functional and network analysis

**Fig 4:**
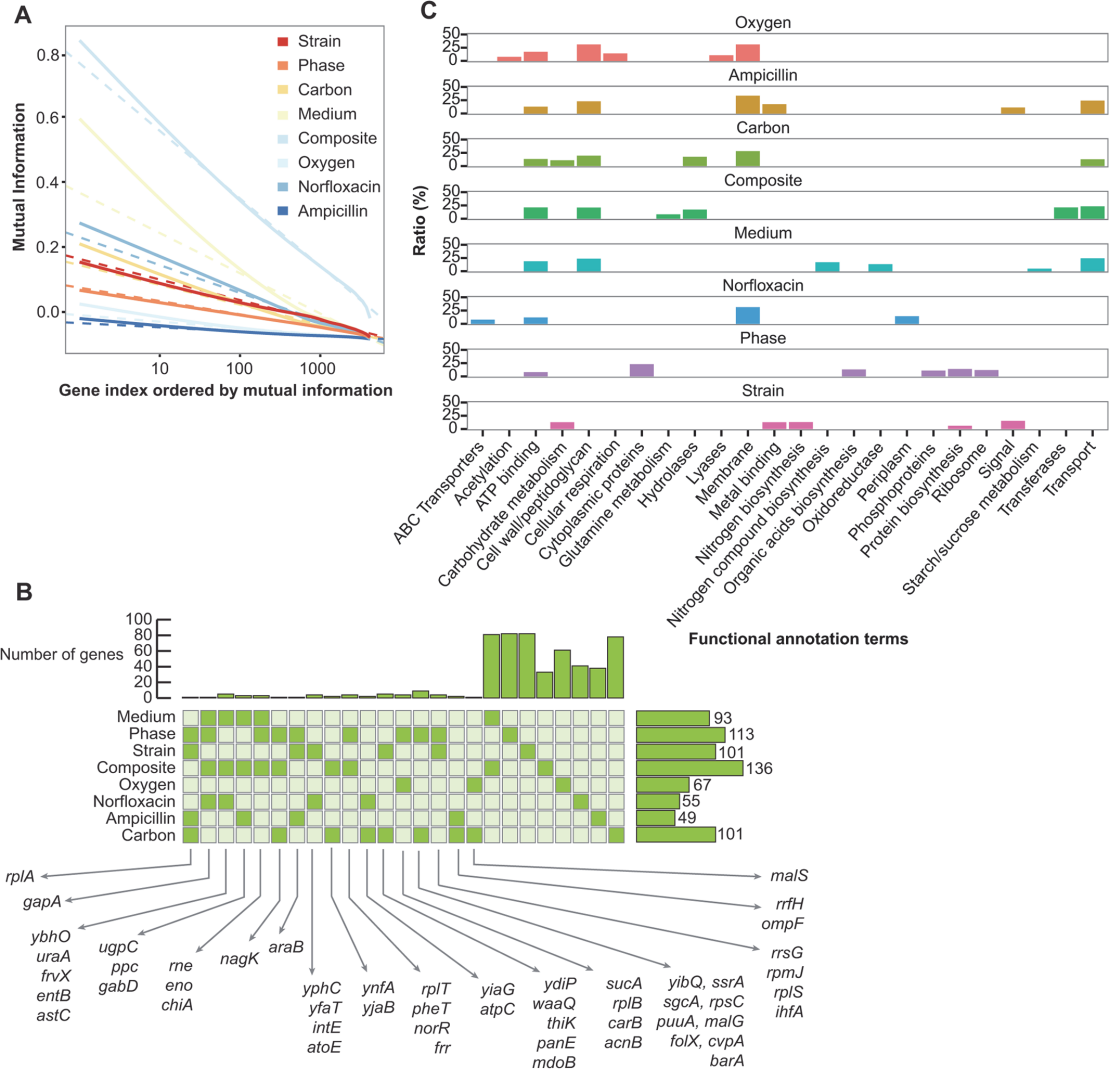
Feature and functional enrichment analysis. **(A)** Mutual information (MI) content for each of the 8 classifiers. The 4166 genes are sorted by decreasing order of their MI. Solid and dashed lines correspond to empirical data and inverse log-linear fitting, respectively. **(B)** The common set of the most informative genes across different classifiers. For each of the 8 classifiers, genes that account for top 10% of MI of all genes are extracted (side bars depict the size of the corresponding gene set). The top histogram depicts the size of the unique features (genes) per classifier. **(C)** Functional annotations of the selected features for each classifier. The six most significantly enriched ontology terms are depicted. As some of functional terms were synonyms, we extract the non-duplicated associated terms. Ratios represent the proportion of the specific ontology terms present in a MI gene set.

**Fig 5:**
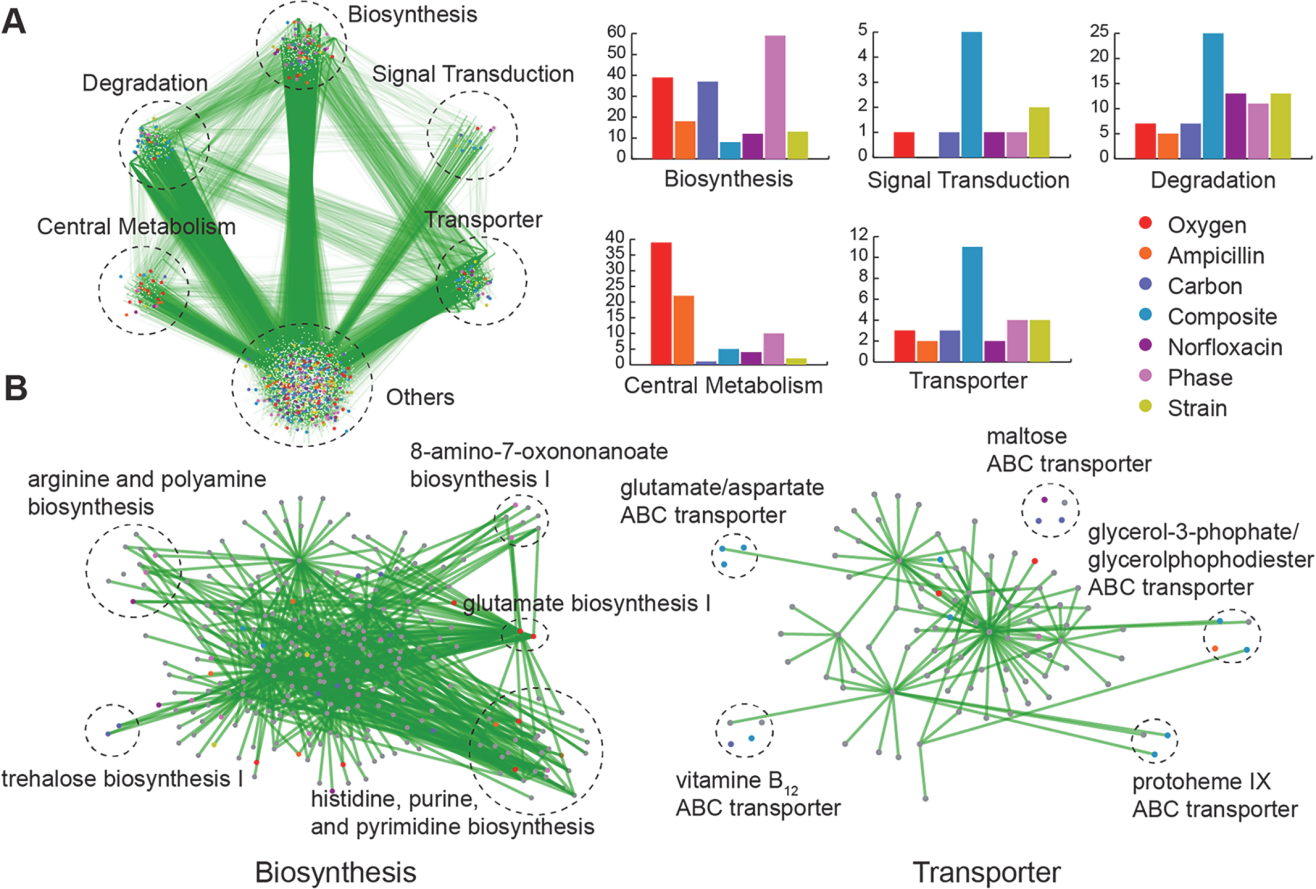
Highly informative genes on a genetic interaction network. **(A)** Genes are grouped into five separate modules that are distinct from the core network. Ontology of pathways and compositions of transporter complexes are based on EcoCyc for *E*. *coli* K-12 MG1655. Green edges represent genetic interactions identified in [47]. Histograms show frequencies of MI genes for different classifiers for 5 pathway modules. **(B)** A higher resolution representation for the biosynthesis and transporter complex pathways that are highly enriched in a number of classifiers. Genes shown are the top-ranked in each classification task. The node color denote the classification task that it is highly informative of (task legend on the upper right of the figure).

Next, we investigated which genes have the highest information content and the respective pathways they belong to. The decrease of mutual information in ranked genes follows an inverse logarithmic relationship (Fig. 4A and S5 Table). For each classifier, we selected the gene subset that accounts for the top 10% of the mutual information content of all genes, yielding feature sets that range from 49 to 136 genes. The overlap among classifiers is substantial: 141 out of a total 715 informative genes (19.7%) are present in two or more different classifiers (Fig. 4B). Functional enrichment analysis of the most informative genes reveals a rich repertoire of biological processes where their differential enrichment is discriminative of each specific class (Fig. 5, S7 Table). Not surprisingly, in the case of the aerobic respiration classifier enriched functional categories include cellular respiration (*P* < 3.1 × 10^−4^). Similarly, for phase and strain classifier, organic acid biosynthesis (*P* < 2.7 × 10^−4^) and nitrogen biosynthesis (*P* < 1.2 × 10^−3^) are up-regulated, respectively. Genes that are related to carbohydrate metabolism (*P* < 6.1 × 10^−7^) are noticeably most informative to classify different carbon sources as well as strains. Some functional characteristics were statistically significant across multiple classifiers, including cell wall/peptidoglycan (*P* < 2.7 × 10^−7^) and ATP-binding (*P* < 1.5 × 10^−8^), hydrolases (*P* < 9.1 × 10^−6^), membrane (*P* < 4.1 × 10^−6^), ribosome (*P* < 2.2 × 10^−7^) and transport (*P* < 4.2 × 10^−6^) (Fig. 4C). The global pathway map in Fig. 5 depicts that most informative genes that were found to belong in five pathway groups: biosynthesis, signal transduction, degradation, transporter and central metabolism. For the composite classification of medium, strain and phase, relevant pathways are implicated with signal transduction, degradation, and transport (Fig. 5A). Moreover, genes for phase classification are enriched in biosynthesis (*P* < 4.3 × 10^−7^) which is in agreement with previous studies that report the prevalence of phase-dependent transcriptional regulation in a variety of biosynthetic processes [37–39]. Fig. 5B provides a more detailed view of the regional network involved in biosynthesis and transport, highlighting the pathways that would be most informative to classify various bacterial characteristics. Highly informative genes involved in specific pathways (e.g. glutamate biosynthesis I, histidine, purine, and pyrimidine biosynthesis and glycerol-3-phophate/glycerol phophodiester ABC transporter) have a crucial role from a functional network perspective, either by being a hub or their first-order neighbors in an identical pathway group.

The analysis of the most informative genes for the media classifier reveals 14 genes encoding for membrane transporters and 7 involved in nitrogen metabolism (Fig. 4, S7A Table, S1 Text). From this set, five are implicated in amino acid transportation and synthesis (*gltK, gltJ, gltL, dppF, glnD*). Different media contain different amounts of amino acids and nutrients required for bacterial growth so the activation of their biosynthesis is expected to be an informative feature about the media where bacteria are growing. Another 3 genes are involved in the enterobactin synthesis (*entA, entE, fepA)*, a siderophore that has been very recently revealed to be related to the growth of *E*. *coli* in M9 [40].

Over the course of the growth curve, the metabolic pathways change in order to optimize the use of the available nutrients and to ensure survival under stress conditions. The major transcriptional regulator for the entry into stationary phase is *RpoS* and, as expected, it is present in the set of genes informative for growth phase, along with several genes belonging to its regulon like *dnaK, clpx, hemL, dps, rpsK, hfq, rplA, crr, rpsE* and *gapA* [41]. In this set of genes, there are also genes already described to be differentially expressed in stationary phase, like *hpf, crr* and *sspA* [42–44]. In addition, ribosomal proteins (*rpsL, rpsQ, rpsE, rplA, rplT, rpmJ, rrsG*) are also implicated to be phase-dependent, which is in agreement with previous reports [45].

In the case of the strain classifier, the analysis displays a wide variety of genes involved in different pathways and cellular processes. Different strains have evolved differentially from their common ancestor and, hence, have developed different regulatory pathways for various processes including carbon assimilation, degradation, and membrane formation. All informative genes for the medium classifier (S7A Table) are included at the top 10% informative genes of the composite classifier with all remaining genes being part of metabolic processes (S7D Table).

Environmental perturbations, such as carbon source and oxygen abundance, give rise to informative genes that are specific to those cellular processes (S7F Table and S7G Table, respectively). In the case of oxygen, GO analysis reveals 8 genes involved in the respiratory process, 4 in aerobic respiration (*sucA, acnB, nuoJ, cyoE*) and another 4 in fermentation (*hycC, hycE, hycF, fhlA*). For carbon source prediction, we can find 15 proteins associated with membrane formation, with 6 of them described transporters (*atpC, kgtP, rhtB, lptG, malF, malG*). In addition, 5 differentially expressed genes involved in carbohydrate metabolism also stand out (*malS, kgtP, malF, malG, pta*).

Regarding antibiotics, we have tested Norfloxacin, which functions by inhibiting DNA gyrase. Unexpectedly, in its informative gene list we cannot find any gene related to DNA repair or SOS response (S7E Table), possibly because these genes are involved also in other environmental conditions and are not antibiotic-specific. Most of the genes that reveal the presence of Ampicillin are membrane proteins and cell wall proteins which is in agreement with its function as cell membrane inhibitor (S7H Table), including the membrane protein porin (*ompF*) that is known to bind ampicillin [46].

Interestingly, a substantial subset of the informative genes that were selected as features were not differentially expressed in the respective samples (S2 Table). A closer look at those genes, which range from 70% to 18% of the corresponding feature set, reveals that they indeed take part in processes that are characteristic of the respective environmental conditions. For instance, the oxygen classifier contains as features genes that are involved in both aerobic (*cyoD, nuoK, sucD, sucC* and *cyoB*) and anaerobic respiration (*hycB, menF, nuoK, nfsA, hypA*), although these genes would not be selected if we ranked based on differential expression. Similarly, in carbon source classification this set includes 11 genes involved in carbohydrate catabolic processes (*dkgB, araG, gatZ, fbaA, malE, murQ, ascF*) and 6 in cellular polysaccharide metabolic processes (*kdsA, kdsD, waaC, waaP, rfaZ, rfa*). The 24 transporters used for the medium classification, the 5 genes involved in translation for phase classification and 72 membrane proteins that are contained in the antibiotic feature set are indeed expected to be informative in the respective classification task, despite not being in the top differentially expressed genes.

### Targeted experimentation of informative genes

The results obtained in this study can be used to decipher novel, condition-specific gene functions. To assess whether biological function can be predicted by targeted experimentation of classifier-specific informative features, we selected one gene with high MI for carbon source classification (*ppiD*) and another gene that is highly ranked for classification between aerobic and anaerobic respiration (*ldcC*). The MI of each gene is only high in the classifier of interest and not in the rest (S8 Table). We then tested knock-out mutants [47] in their respective conditions. As such, both the *ppiD* and *ldcC* mutants and the wild type strain were grown in M9 supplemented with three different carbon sources: glucose, glycerol and lactate. The *ldcC* mutant functions as a negative control in the case of carbon source classification since this mutation is expected to have no effect on medium determination. Indeed, the results (S3 Fig., S12 Table) show that Δ*ppiD* growth is impaired in the presence of the three sugars (*t* – *test, P* < 0.03) while growth with the *ldcC* mutant remains similar to the WT demonstrating the involvement of *ppiD* in the use of different carbon sources (*t* – *test, P* > 0.07). *ppiD* has been described as a membrane-anchored chaperone [48] but its specific function has not been discovered. Our result suggest that this protein is involved in sugar metabolism, possibly related to folding activity of membrane sugar transporters. Growth curves for knockout replicates of the top five informative genes for different carbon sources, as well as the growth curves for the genes related to aerobic growth genes (as negative control), are shown in S4 Fig.. As expected, growth deficits were more pronounced in the first set in both glycerol and lactate (*t* – *test, P* < 0.006 and *P* < 0.008, respectively).

We performed a similar experiment where the three strains (WT, Δ*ppiD* and *ΔldcC*) *were* grown in M9 with glucose in aerobic and anaerobic conditions, in order to assess the influence of the *ldcC* mutation in these conditions. Here, the *ppiD* mutant serves as the negative control and the *ldcC* mutation is indeed informative of the aerobic conditions, although the difference is not as pronounced as in the case of carbon source classification (*P* < 0.029 for *ppiD*; *P* > 0.080 for *ldcC*). A closer look at the MI values show that the informative genes for aerobic respiration are two orders of magnitude lower than those for medium, which suggests that information content is dispersed among a number of genes.

## Discussion

How much information regarding the life and the present environmental context can be inferred from the global transcription profile of an organism? To address this question, we constructed an extensive, annotated gene expression compendium, where we trained Bayesian models for seven distinct classification tasks. Our models achieved high classification performance that was robust on the number of genes that were used as informative features. Our work demonstrates that bacterial transcriptomes embody rich information regarding the organism and the environment that it inhabits. Recent work demonstrates the power of such datasets to identify data-driven ontologies and rethink the definition of biological processes within them [49]. More importantly, multiple characteristics of an organism can be accurately predicted using a set of character-specific classifiers, suggesting practical advantages of this approach over limited datasets.

Transcriptional activity is not the sole feature type that conveys predictive information regarding environmental conditions and an organism’s characteristics. Like eukaryotes, epigenetic signals regulate transcriptional activity in bacteria, for example, by altering DNA methylation states to control the binding of proteins to DNA [50]. Single-molecule real-time (SMRT) sequencing technology has been recently applied to reading of genome-scale methylation states in a pathogenic *E*. *coli* [51] and the technology would provide higher-resolution of molecular information of bacteria, enabling fine-scale predictive characterization based on it. Other features related to the genome-scale metabolic state, proteomic biomarkers and cell morphology can be incorporated to increase the predictive capacity of any given classifier. Similarly, while the six characteristics that we evaluated here are fundamental in their role and indicative of global processes, there are several other environmental and organismal characteristics, such as other abiotic factors or other microbial species in the same environment, which can be predicted from these features.

Multiple characteristics of an organism are interrelated, implying its heterologous transcriptional landscapes in different combinations of phenotypic conditions. These complex dependencies in phenome are not readily analyzable even in the compilation of thousands of publicly available transcriptome profiles as the experimental conditions in published data are often disproportionate, typically skewed in favorable settings (e.g. MG1655 strain over LB medium), which produces small sample sets or even empty sets in combinatorial conditions. Indeed, the results on composite classification argues that with the current *omics* dataset compilation, it is not feasible to explore many of the strain, phase, medium combinations, as we have sufficient data for only 13 classes, out of a total of 48 possible classes (4 for each of medium, phase, strain). Interestingly, the performance of the composite multi-class classifier performs significantly better for the overall classification of these characteristics, than an aggregate of individual classifiers for phenotypes, demonstrating large interdependencies across different conditions.

By looking at the top informative genes in two classifiers, we demonstrated the involvement of the *ppiD* in the utilization of different carbon sources. Further analysis involves the use of over/under-expressed copies and protein-protein assays to discover quantitative associations and interaction partners. By analyzing the expression levels of the genes in the phase classifier that are not predictable using RT-PCR and transcriptional fusions we can find out novel regulation when growth phase changes from exponential to stationary. Another potential application is in the case of the antibiotics Ampicillin and Norfloxacin where this analysis can be used to identify implicated pathways in lethal and non-lethal concentrations.

In recent years, the capacity of microorganisms to sense and act upon environmental stimuli [52] has sparked renewed interest due to its diverse applications in preventive medicine and synthetic biology [53,54]. These studies shed light on the adaptive behavior of cells under environmental temporal stimuli [55–57] and on the decomposition of promoter activity in complex conditions [58]. Our work here is the first that attempts to identify and comprehensively interpret the capacity of the transcriptome for characterizing a manifold of environmental conditions using the consensus of multiple statistical learning algorithms. Aside from its intellectual merit, the presented work can help building classifiers and selecting features in a number of practical applications. Detection and characterization of microbes are of great importance in many clinical, environmental, industrial, and agricultural application [59]. Data are increasingly become available for the adoption of such classification techniques since high-throughput methods have been recently applied at low cost. From battlefields to agricultural crop management, inexpensive sequencing transforms the landscape of what is possible in a timely, inexpensive manner. Our work paves the way towards the use of high-throughput expression datasets to a broad range of applications including detection and characterization of the environmental conditions and bacterial population that are important for clinical, environmental, industrial, and agricultural applications. Without loss of generality, this work can be described as a data-driven approach to “bacterial forensics”, i.e. the extraction of environmental knowledge from large-scale phenotypic bacterial data, and it can have far-reaching applications in environments that would be challenging to investigate otherwise.

## Methods

### Construction of a microarray and RNA-Seq compendium (EcoGEC)

We downloaded 83 RNA-Seq *E*. *coli* transcriptional profiles from 17 different GEO entries [30] that correspond to 8 strains, LB and MOPS media in wild-type (WT), gene knock-outs (KOs), double KOs and environmental perturbations. When bedGraph format was used in the data, gene expression level was measured in RPKM using the bgrQuantifier program that is part of the RSEQ tool [60]. For other formats such as wig, we first converted them into bedGraph. We filtered out samples where the environmental information was not known, which led to 64 samples for further analysis. Data were converted to log2 scale and performed quantile-normalization using MATLAB. The resulting RNA-Seq dataset was composed of 64 samples of 4725 genes. We integrated the RNA-Seq dataset (64 samples) to the *E*. *coli* Microarray Compendium (EcoMAC) that consists of 2198 microarrays of 4189 genes for which raw files were downloaded and normalized by RMA (robust multichip average) method [29]. The integrated EcoGEC dataset consists of 2262 samples and 4166 genes (Fig. 1A, S13 Table, S14 Table).

### Adjustment of batch-effects in the transcriptome compendium

Although integrative analysis of multiple microarray gene expression (MAGE) datasets allows to distill the maximum relevant biological information from genomic datasets, the unwanted variation, so-called batch-effects arising from data merged from difference sources has been a major challenge to impede such effort [61]. To adjust the non-biological experimental variation with the consideration of large number of datasets with a few samples, we used ComBat that is developed under Bayesian framework and is known to be robust to outliers in small sample sizes [62]. In the process of adjustment, we took into account experimental conditions as covariates to prevent loss of biological variations.

### Categorization of gene expression data

Prior to building a prediction model, we transformed the adjusted gene expression data into categorical values (under-expressed, UE; wild-type, WT; over-expressed, OE) in order to deal with biases arising from combining different platforms and improve the classification accuracy [63]. We first measured the log_2_ *Fold Change* (*FC*) of gene expression with respect to the WT expression for each gene. WT samples were identified from experiments that didn’t undergo genetic and environmental perturbations from the three platforms (7 for Affymetrix *E*. *coli* Antisense Genome Array, 6 for Affymetrix *E*. *coli* Genome 2.0 Array, and 6 for RNA-Seq). log_2_ *Fold Change* (*FC*) was separately measured for each platform by comparing the mean of WT data. Using transformed data, we estimated a normal distribution *N*(*μ, σ* ^2^) for each gene and finally converted each log_2_ *FC* gene value into one of the 3 categorical values by measuring deviation from the mean (UE when *g*_*ij*_ < *μ*_*i*_ – *σ*_*i*_; WT when *μi* – *σ*_*i*_ ≤ *g*_*ij*_ ≤ *μ*_*i*_ + *σ*_*i*_; OE when *μ*_*i*_ + *σ*_*i*_ *< gij; g*_*ij*_ is the log_2_ *FC* for gene *i* in sample *j, μ*_*i*_ is mean of gene *i* and *σ*_*i*_ is standard deviation of gene *i*). The platform-specific categorization of gene expression effectively removes platform biases (Fig. 1B).

### Inference of missing phase information using iterative learning

The large fraction of unannotated phase data in the compendium hinders the maximum utilization of such resource. Missing phase information was imputed by iterative learning approach in which prediction model for growth phase is trained using the annotated phase data and inferred data in previous iteration until prediction of unknown data finally reaches at convergence (S1 Fig.). In each iteration, the experiments that were unannotated *ab initio* were repeatedly inferred. Inference is based on consensus-based approach of four machine learning methods described above. Re-labeled phase information accompanying with annotated data is used for training the consensus model in next iteration. This procedure is halted once the similarity of phase labels between consecutive iterations is convergent when the similarity of phase labels between consecutive iterations converges (change in fraction < ξ, where ξ = 0.01 here). Although the use of inferred labels through iterative learning demonstrates an increased performance, compared with the prediction using known labels only (S2 Table), we report the performance for phase prediction using annotated labels only throughout the manuscript.

### Validation of iterative learning

To investigate the accuracy (balanced) of inference of unannotated data, we performed the simulation study for each classifier by randomly masking 30% of total labels of each class. First, the accuracy of inferred annotation after iterative learning is measured by comparing with real labels before and after iterative learning (S2 Table). Then we further evaluate iterative learning for by changing the percentage of unannotated labels (2%, 5%, 10%, 20%) in the total data (S2 Fig. and S4 Table).

### Class labeling

A label is assigned for each of the seven classification characteristics (two for antibiotics; Ampicillin and Norfloxacin). We have identified 4 classes for medium (LB, M9, MOPS, others), 3 classes for phase (early-exponential, mid/late-exponential, stationary) having both annotated and predicted data, and 4 classes for strain (MG1655, BW25113, EMG2, others). A class of “others” was added that corresponds to conditions that are unclear or scare in quantity. Classification of the strain, medium, and growth conditions can be integrated also as a multi-class problem. We synthesized a new predictor variable called composite by combining values of 3 characteristics. From the 48 possible classes (combination of 4 labels for medium, 3 labels for phase, 4 labels for strain), only 13 combinations have enough data (more than 5 samples) for training, hence we have encompass all other labels with insufficient data under the label “others”, resulting in a total of 13 classes (Table 2).

### Consensus-based predictions

We use Naïve Bayes (NB; [64], Decision Trees (DT, [65]), K-nearest-neighbors (KNN, [66]) and Support Vector Machines (SVM, [67]) to construct a consensus classification scheme [68]. The class label assigned is the one with the highest number of votes. The predictive power is assessed through Receiver-Operator Characteristic (ROC) and Precision-Recall (PR) curves [36]. For multi-class problems, such as in the case of medium, phase and strain classification, we built ROC/PR curves in a one-versus-rest (OVR) approach.

The leave one batch out cross validation was conducted to verify model performance while removing batch effects. For this, each batch is left out for testing and the rest of data is then used for training. This procedure is iterated until all batches in the dataset are tested. For carbon source, phase, and composite classifier, the profiles having early-exponential phase or acetate are studied in a single project so inevitably, we had to rely on the batch-uncontrolled cross-validation. The classifier performances with and without batch control are compared in S11 Table. As the high imbalance of class distribution is observable in the dataset as shown in Table 1, creating inflated baseline, we show the classifier performance for the original dataset as well as for the dataset with balanced class distribution.

### Feature selection by mutual information

Mutual information is a stochastic measure of dependence [69] and it has been widely applied in feature selection in order to find an informative subset for model training [70]. In our work, each of the eight models were trained with the top *k*-ranked genes based on their mutual information (MI) to the label where MI is measured by

$$I(X;Y)=\sum_{}^{}\sum_{}^{}p(x,y)\log(p(x,y)/p(x)p(y))$$

Where *x* is the gene selected and *y* is the predictor variable. This process is iteratively repeated by increasing *k* with an interval of 10 and the exception of start (10) and end points (all genes). Basically, the selection procedure of *k* features are performed in training data only and *k* showing the highest performance is selected for testing. All the analyses in this study other than the cross-validation of model used the features selected from the complete data.

### Selection of most informative genes and functional enrichment analysis

The most informative genes are selected by measuring the mutual information (in bits) for each of the characteristic variables and then selecting the top 10% genes based on their information content. These top informative genes are then used for finding shared genes across different classifiers (Fig. 4B) and for network analysis (Fig. 5). For functional enrichment analysis, we use all selected genes that optimize the classifier performance. Associated functional annotations for the set of selected genes for each of the classifiers are found by DAVID [71]. Various annotations including Gene Ontology terms, KEGG pathways, and InterPro protein domains are investigated. Among them, the 6 most statistically significant terms (*P* < 3.7 10^−4^) for each classifier are displayed in Fig. 4. Global map of genetic interactions for *E*. *coli* is reconstructed from [72] with pathway modules that functionally cluster genes based on the Pathway Ontology and transporter complexes curated in EcoCyc [73]. Pathway diagrams were re-plotted from the KEGG database [74].

In addition to DAVID, we have performed a GSEA analysis [75] where each gene is ranked by its mutual information (S9 Table). We have also compared the results to those obtained by DAVID and provide this comparison in S10 Table. On average, 80.5% of DAVID results that correspond to the feature set at optimal classification performance are in the GSEA enriched terms.

### Growth curves

Growth curves of the WT, Δ*ppiD* and Δ*ldcC* were performed in M9 complemented with 0.4% of glucose, glycerol and sodium lactate. For growth curves, the starter cultures of all strains were grown and therefore adapted (B7–9 generations) to M9 glucose for 12 hours at 37C. Cultures were started at OD600 of 0.004. OD600 was measured every 10 minutes on a Tecan Plate Reader. Two independent replicate growth tests were performed for each strain. For the anaerobic and aerobic growth curves bacteria were grown in M9 supplemented with glucose at 37C without shaking. The anaerobic growth was made in an anaerobic chamber where media was inserted 2 days prior to the experiment to extract all the oxygen present in the media. Samples were taken at 2, 8 and 24 hours through a spectrophotometer (S12 Table).

### Parameter settings, implementation, and availability

For consensus-based prediction using four different classifiers, we used the Statistics Toolbox in MATLAB. For the multi-class SVM, one-versus-rest (OVR) approach was used in which for each class, a binary classifier is built for the class label and the rest. Each binary SVM was built using Gaussian Radial Basis Function (RBF) kernel and the default sigma factor of 1 was used. For soft margin, C parameter showing best performance was selected in the range of 0.5 to 4 in the training phase. For KNN, K was set to one in *knnsearch*. For decision tree and naïve Bayes, the default settings in *ClassificationTree* and *NaiveBayes* were used, respectively. The code used in this study including the imputation by iterative learning and the consensus-based prediction that allows users to reproduce the results is freely available on gitHub (https://github.com/minseven/mForensics.git).

## Supporting Information

S1 Fig Schematic diagram of iterative learning for phase information. Missing phase information was imputed by an iterative learning approach in which the prediction model for growth phase is trained iteratively until convergence. In each iteration, the phase of all samples that were originally unannotated is predicted, based on an ensample of 4 machine learning methods (Naive Bayes, SVM, Decision Tree, KNN) that produce a consensus outcome, as described in the Methods section of the manuscript. We used the fraction of correctly re-annotated data over all unannotated data to measure the similarity of the two vectors. If the confidence level of the prediction does not reach a threshold of 0.75 (i.e. 3 out of the 4 methods agree in the putative annotation), then the sample remains tagged as un-annotated. Otherwise, the imputed phase information is used for training the model during the next iteration. This procedure repeats until the similarity of phase labels between consecutive iterations converges (change in fraction < ξ, where ξ = 0.01 here). For our dataset, this led to annotation of more than 90% of the un-annotated data samples.
(EPS)

S2 Fig Validation of iterative learning and imputation of unannotated phase data. (A) Prediction of unknown phase information by using the iterative approach is validated by using testing data that consist of de-labeled samples constructed from each of the 3 phase types (early exponential, mid/late exponential and stationary). Validation of iterative approach to impute missing data is performed by comparing the actual and predicted labels produced by iterative learning. The de-labeled, i.e. artificially un-annotated, data were 2%, 5%, 10% and 20% of the total dataset. For each of the 3 phase classes, the predicted classes for each actual class type is shown. (B) Unannotated portion of phase data in the EcoGEC is inferred by using iterative learning. After four iterations, the similarity of predicted labels between consecutive iterations converge (691 out of the 764 samples). The 72 leftover samples are discarded as unidentified and/or noisy data points. (C) Simulation of iterative learning for all classifiers by randomly masking 30% of all class labels in the original dataset. We set the threshold of confidence of consensus-based prediction to 1 for selecting data that needs to go to next iteration over the iterative learning. In other words, the samples that reach perfect consensus in assigning labels from 4 different methods are finalized for annotation and used for training over the iterative learning. The purpose of the more stringent threshold was to observe the benefit of iterative process in learning. The percentages in the legend indicate the total increase of re-labeled classes after the first iteration.
(EPS)

S3 Fig Targeted experimentation of highly informative genes. (A) Growth curves of WT, Δ*ppiD* and Δ*ldcC* for the three carbon source classes in our dataset, glucose, glycerol and sodium lactate, (B) growth curves of WT, Δ*ppiD* and Δ*ldcC* in aerobic and un-anaerobic conditions.
(TIFF)

S4 Fig Growth curves of the five most informative genes in the carbon source classifier (left) and oxygen (right) in M9 salt media supplemented with three different carbon sources. Each growth curve was made in duplicate and the average was plotted.
(TIFF)

S1 Table Classifier performance and sample size. The relationship between performance of classifiers and the data size is investigated. The dataset with balanced class distribution is prepared from the original compendium and it is reduced by 25% until only 25% remains. Each dataset is separately trained and tested.
(XLSX)

S2 Table Comparison of classification performance between classifiers with the top MI genes and DE genes. The intersection of the feature gene set when mutual information (MI) and differential expression (DEG) are used for ranking. Differential expression ranking was determined by ANOVA. In the parenthesis, we report the classification performance when the class labels are uniformly distributed (maximum entropy). The “null” and “dataset” baselines correspond to the base prediction accuracy in the case where the classes are uniformly distributed for each classification task, or the most representative class based on the data (highest prior) for each classification case is selected, respectively.
(XLSX)

S3 Table Evaluation of iterative learning on classification performance. We assessed the iterative learning (IL) method for each class by randomly masking 30% of the class labels (testing dataset). Accuracy refers to the percentage of the testing dataset that was correctly re-annotated by IL. Classification performance is measured with and without IL being applied to the final dataset. The “null” and “dataset” baselines correspond to the base prediction accuracy in the case where the classes are uniformly distributed for each classification task, or the most representative class based on the data (highest prior) for each classification case is selected, respectively.
(XLSX)

S4 Table Performance of iterative learning and unannotated proportion of phase data. To evaluate the efficacy of iterative learning to correctly annotate missing phase information, we constructed a testing set where data for all 3 different phase categories (early-exponential, mid/late exponential, and stationary) were de-labeled. We evaluated how many samples in this simulated, artificially un-annotated, dataset were re-labeled to their original phase labels after iterative learning. The iterative learning procedure was performed with testing sets that included 2%, 5%, 10% and 20% of un-annotated samples, as a percentage of all samples available. The tables below (1A-D) show that the contingency tables of simulated inference for different settings.
(XLSX)

S5 Table Ranked list of all genes in the EcoGEC compendium based on their mutual information for the phase, growth and aerobic classifier, before and after iterative learning. (XLSX)

S6 Table Contingency table of prediction performance for each classifier. (XLSX)

S7 Table List of most informative genes annotated with known functions. (XLSX)

S8 Table Ranks and mutual information of the genes selected in each classifier of carbon source and oxygen supply. (XLSX)

S9 Table GSEA enriched terms: enriched terms in red are present in DAVID analysis. (XLSX)

S10 Table Proportion of DAVID results in GSEA enriched terms. (GO). For DAVID, we use all selected features for each classifier to know associated GO. For GSEA, we use MI of all genes to know enriched GO. The resulting lists are compared and the proportion of DAVID results in GSEA enriched terms are reported.
(XLSX)

S11 Table Classification performance with and without batch control. The prediction performance with and without batch control is compared for each classifier. For carbon source, phase, and composite classifier, the profiles having early-exponential phase or acetate are studied in a single project. For testing without batch control, typical 5-fold cross-validation was used without considering the batch information. For batch controlled experiments, a leave-one-batch-out cross validation was conducted to verify model performance while removing batch effects.
(XLSX)

S12 Table Measured growth data. (XLSX)

S13 Table EcoGEC v1.0 Compendium (part 1). (XLSX)

S14 Table EcoGEC v1.0 Compendium (part 2). (XLSX)

S1 Text Reference list of S7 Table. The citations on the functional studies of the ranked list of genes in S7 Table are listed.
(DOCX)
