# Correction: Microbial Forensics: Predicting Phenotypic Characteristics and Environmental Conditions from Large-Scale Gene Expression Profiles

**DOI:** 10.1371/journal.pcbi.1004617

**Published:** 2015-11-20

**Authors:** The PLOS Computational Biology Staff

Table 1 is incorrect. The numbers for "Class Labels" in "Nor" column should be 79 and 2163 for Y and N, respectively. The numbers for "Class Labels" in "Amp" column should be 227 and 2015 for Y and N, respectively.

Please view the correct table here:

**Table 1:** Class label distributions

	Classes													
	Medium		Strain		Phase		Oxygen		Nor		Amp		Carbon	
**ClassLabels**	LB	1356	MG1655	1368	E-Exp	148	Y	2178	Y	79	Y	227	Glucose	471
	M9	301	BW25113	148	ML-Exp	1368	N	64	N	2163	N	2015	Glycerol	94
	MOPS	86	EMG2	132	Stat	132							Acetate	49
	Others	499	Others	594	Missing	601							Others	1628
**Baseline**	60.4%		61%		61%		97.1%		96.3%		89.8%		72%	
