# Supplementary figures and images for: Microbial Forensics: Predicting Phenotypic Characteristics and Environmental Conditions from Large-Scale Gene Expression Profiles
Source: PLoS Comput Biol. 2015 Mar 16;11(3):e1004127. doi: 10.1371/journal.pcbi.1004127 (PMC4361189; doi:10.1371/journal.pcbi.1004127)

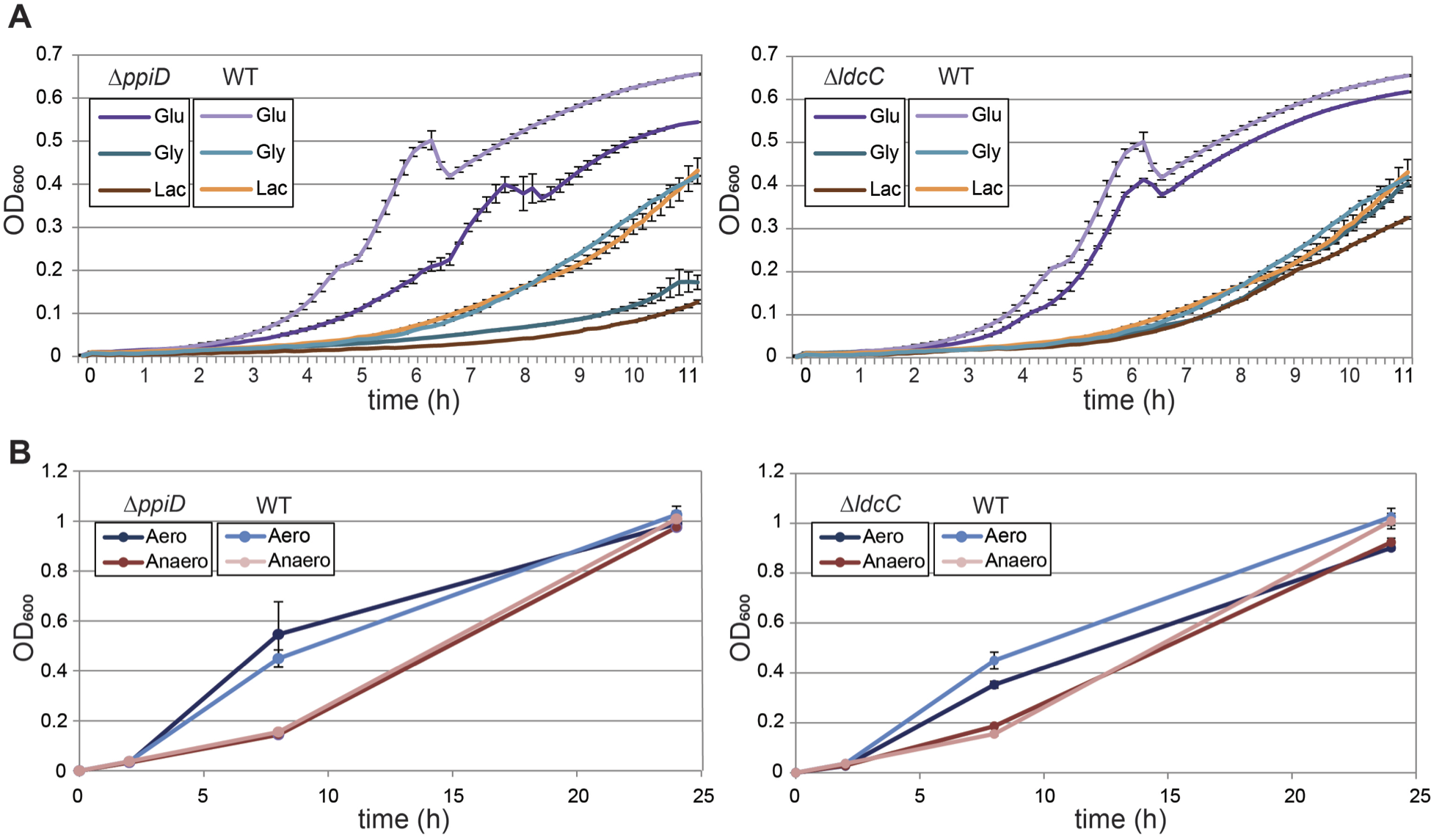

Supplement: S3 Fig — (A) Growth curves of WT, ΔppiD and ΔldcC for the three carbon source classes in our dataset, glucose, glycerol and sodium lactate, (B) growth curves of WT, ΔppiD and ΔldcC in aerobic and un-anaerobic conditions. (TIFF) [file pcbi.1004127.s003.tiff]

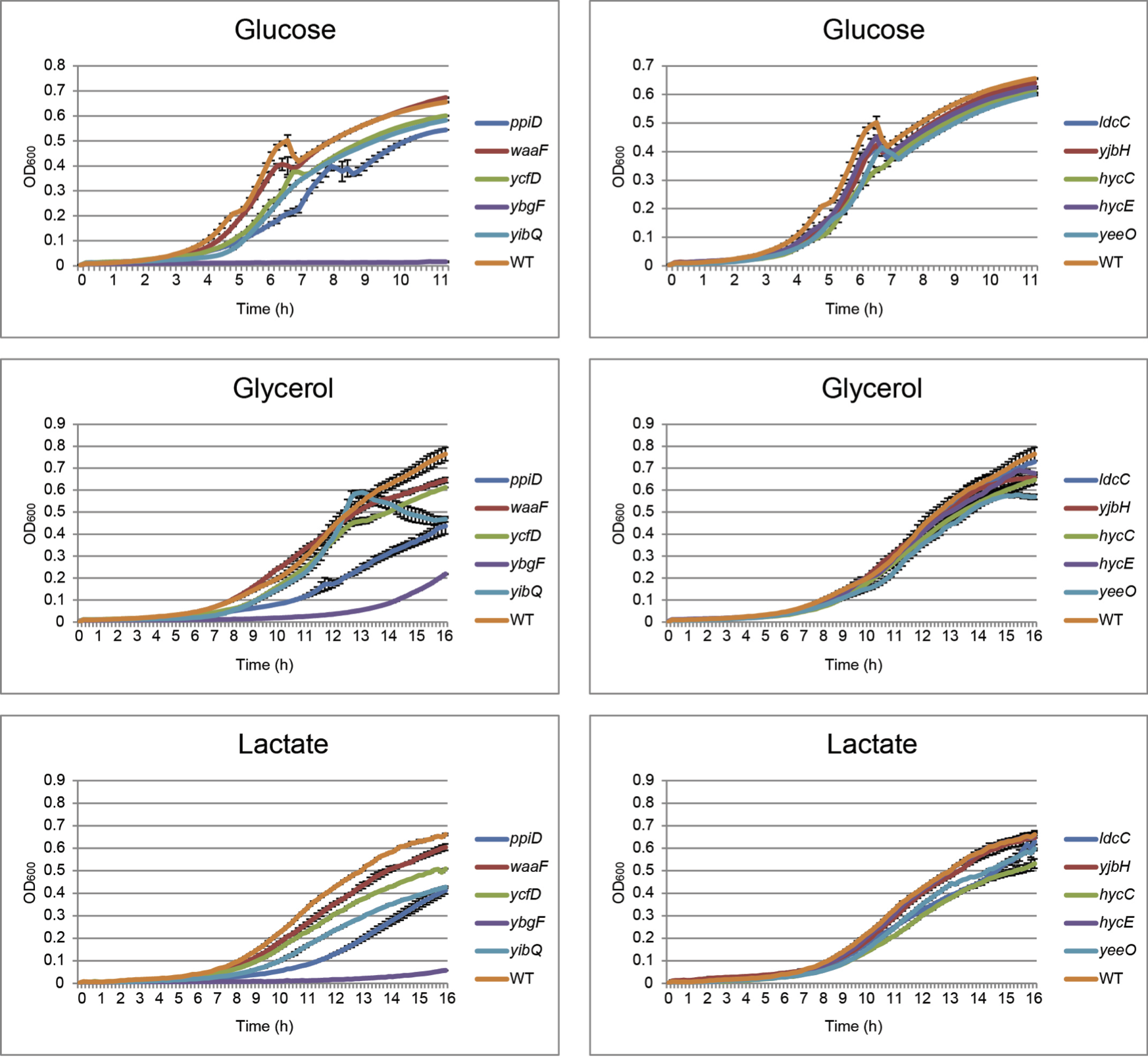

Supplement: S4 Fig — Each growth curve was made in duplicate and the average was plotted. (TIFF) [file pcbi.1004127.s004.tiff]
