# Supplementary material for: Microbial Forensics: Predicting Phenotypic Characteristics and Environmental Conditions from Large-Scale Gene Expression Profiles
Source: PLoS Comput Biol. 2015 Mar 16;11(3):e1004127. doi: 10.1371/journal.pcbi.1004127 (PMC4361189; doi:10.1371/journal.pcbi.1004127)
Supplement: S1 Text — The citations on the functional studies of the ranked list of genes in S7 Table are listed. (DOCX) [file pcbi.1004127.s019.docx]

**Supporting Reference List**

[1] Edwards MD, Black S, Rasmussen T, Rasmussen A, Stokes NR, et al. (2012) Characterization of

three novel mechanosensitive channel activities in *Escherichia coli*. Channels (Austin) 6: 272-281.

[2] Liu J, Duncan K, Walsh CT (1989) Nucleotide sequence of a cluster of *Escherichia coli* enterobactin

biosynthesis genes: identification of entA and purification of its product 2,3-dihydro-2,3-

dihydroxybenzoate dehydrogenase. J Bacteriol 171: 791-798.

[3] Zogaj X, Nimtz M, Rohde M, Bokranz W, Romling U (2001) The multicellular morphotypes of

Salmonella typhimurium and *Escherichia coli* produce cellulose as the second component of the

extracellular matrix. Mol Microbiol 39: 1452-1463.

[4] Adler C, Corbalan NS, Peralta DR, Pomares MF, de Cristobal RE, et al. (2014) The alternative role of

enterobactin as an oxidative stress protector allows *Escherichia coli* colony development. PLoS One 9:

e84734.

[5] Izu H, Adachi O, Yamada M (1997) Gene organization and transcriptional regulation of the gntRKU

operon involved in gluconate uptake and catabolism of *Escherichia coli*. J Mol Biol 267: 778-793.

[6] Staab JF, Elkins MF, Earhart CF (1989) Nucleotide sequence of the *Escherichia coli* entE gene. FEMS

Microbiol Lett 50: 15-19.

[7] Dermic D, Halupecki E, Zahradka D, Petranovic M (2005) RecBCD enzyme overproduction impairs

DNA repair and homologous recombination in *Escherichia coli.* Res Microbiol 156: 304-311.

[8] Verkamp E, Backman VM, Bjornsson JM, Soll D, Eggertsson G (1993) The periplasmic dipeptide

permease system transports 5-aminolevulinic acid in *Escherichia coli*. J Bacteriol 175: 1452-1456.

[9] Gehring AM, Mori I, Walsh CT (1998) Reconstitution and characterization of the *Escherichia coli*

enterobactin synthetase from EntB, EntE, and EntF. Biochemistry 37: 2648-2659.

[10] Atkinson MR, Ninfa AJ (1998) Role of the GlnK signal transduction protein in the regulation of

nitrogen assimilation in *Escherichia coli*. Mol Microbiol 29: 431-447.

[11] Pistocchi R, Kashiwagi K, Miyamoto S, Nukui E, Sadakata Y, et al. (1993) Characteristics of the

operon for a putrescine transport system that maps at 19 minutes on the *Escherichia coli* chromosome. J

Biol Chem 268: 146-152.

[12] Klein M, Sprenger GA, Freudl R (1996) Cloning, nucleotide sequence, and functional expression of

the *Escherichia coli* enolase (eno) gene in a temperature-sensitive eno mutant strain. DNA Seq 6: 351-

355.

[13] Reuven NB, Koonin EV, Rudd KE, Deutscher MP (1995) The gene for the longest known *Escherichia*

*coli* protein is a member of helicase superfamily II. J Bacteriol 177: 5393-5400.

[14] Checroun C, Gutierrez C (2004) Sigma(s)-dependent regulation of yehZYXW, which encodes a

putative osmoprotectant ABC transporter of *Escherichia coli.* FEMS Microbiol Lett 236: 221-226.

[15] Jiang P, Peliska JA, Ninfa AJ (1998) Enzymological characterization of the signal-transducing

uridylyltransferase/uridylyl-removing enzyme (EC 2.7.7.59) of *Escherichia coli* and its interaction with the

PII protein. Biochemistry 37: 12782-12794.

[16] Kiino DR, Rothman-Denes LB (1989) Genetic analysis of bacteriophage N4 adsorption. J Bacteriol

171: 4595-4602.

[17] Ow MC, Liu Q, Mohanty BK, Andrew ME, Maples VF, et al. (2002) RNase E levels in *Escherichia coli*

are controlled by a complex regulatory system that involves transcription of the rne gene from three

promoters. Mol Microbiol 43: 159-171.

[18] Yeom JH, Shin E, Go H, Sim SH, Seong MJ, et al. (2008) Functional implications of the conserved

action of regulators of ribonuclease activity. J Microbiol Biotechnol 18: 1353-1356.

[19] Wanner BL (1992) Genes for phosphonate biodegradation in *Escherichia coli.* SAAS Bull Biochem

Biotechnol 5: 1-6.

[20] Yang K, Wang M, Metcalf WW (2009) Uptake of glycerol-2-phosphate via the ugp-encoded

transporter in *Escherichia coli* K-12. J Bacteriol 191: 4667-4670.

[21] Yamada M, Makino K, Amemura M, Shinagawa H, Nakata A (1989) Regulation of the phosphate

regulon of *Escherichia coli*: analysis of mutant phoB and phoR genes causing different phenotypes. J

Bacteriol 171: 5601-5606.

[22] Simon G, Mejean V, Jourlin C, Chippaux M, Pascal MC (1994) The torR gene of *Escherichia coli*

encodes a response regulator protein involved in the expression of the trimethylamine N-oxide reductase

genes. J Bacteriol 176: 5601-5606.

[23] Park JT, Shim JH, Tran PL, Hong IH, Yong HU, et al. (2011) Role of maltose enzymes in glycogen

synthesis by *Escherichia coli*. J Bacteriol 193: 2517-2526.

[24] Diges CM, Uhlenbeck OC (2005) *Escherichia coli* DbpA is a 3' --> 5' RNA helicase. Biochemistry 44:

7903-7911.

[25] Nachin L, Nannmark U, Nystrom T (2005) Differential roles of the universal stress proteins of

*Escherichia coli* in oxidative stress resistance, adhesion, and motility. J Bacteriol 187: 6265-6272.

[26] Guillier M, Allemand F, Graffe M, Raibaud S, Dardel F, et al. (2005) The N-terminal extension of

*Escherichia coli* ribosomal protein L20 is important for ribosome assembly, but dispensable for

translational feedback control. RNA 11: 728-738.

[27] Ikegami A, Nishiyama K, Matsuyama S, Tokuda H (2005) Disruption of rpmJ encoding ribosomal

protein L36 decreases the expression of secY upstream of the spc operon and inhibits protein

translocation in *Escherichia coli.* Biosci Biotechnol Biochem 69: 1595-1602.

[28] Bukau B, Horwich AL (1998) The Hsp70 and Hsp60 chaperone machines. Cell 92: 351-366.

[29] Albrecht R, Zeth K (2011) Structural basis of outer membrane protein biogenesis in bacteria. J Biol

Chem 286: 27792-27803.

[30] Withey JH, Friedman DI (2003) A salvage pathway for protein structures: tmRNA and trans-

translation. Annu Rev Microbiol 57: 101-123.

[31] Polikanov YS, Blaha GM, Steitz TA (2012) How hibernation factors RMF, HPF, and YfiA turn off

protein synthesis. Science 336: 915-918.

[32] Craigen WJ, Cook RG, Tate WP, Caskey CT (1985) Bacterial peptide chain release factors:

conserved primary structure and possible frameshift regulation of release factor 2. Proc Natl Acad Sci U S

A 82: 3616-3620.

[33] Galloway SM, Raetz CR (1990) A mutant of *Escherichia coli* defective in the first step of endotoxin

biosynthesis. J Biol Chem 265: 6394-6402.

[34] Burton BM, Baker TA (2005) Remodeling protein complexes: insights from the AAA+ unfoldase ClpX

and Mu transposase. Protein Sci 14: 1945-1954.

[35] Holbrook EL, Greene RC, Krueger JH (1990) Purification and properties of cystathionine gamma

synthase from overproducing strains of *Escherichia coli*. Biochemistry 29: 435-442.

[36] Galli E, Gerdes K (2010) Spatial resolution of two bacterial cell division proteins: ZapA recruits ZapB

to the inner face of the Z-ring. Mol Microbiol 76: 1514-1526.

[37] Gerhart J (2014) From feedback inhibition to allostery: the enduring example of aspartate

transcarbamoylase. FEBS J 281: 612-620.

[38] Vollmer W, von Rechenberg M, Holtje JV (1999) Demonstration of molecular interactions between

the murein polymerase PBP1B, the lytic transglycosylase MltA, and the scaffolding protein MipA of

*Escherichia coli.* J Biol Chem 274: 6726-6734.

[39] van Heeswijk WC, Molenaar D, Hoving S, Westerhoff HV (2009) The pivotal regulator GlnB of

*Escherichia coli* is engaged in subtle and context-dependent control. FEBS J 276: 3324-3340.

[40] Baglieri J, Beck D, Vasisht N, Smith CJ, Robinson C (2012) Structure of TatA paralog, TatE,

suggests a structurally homogeneous form of Tat protein translocase that transports folded proteins of

differing diameter. J Biol Chem 287: 7335-7344.

[41] Altuvia S, Almiron M, Huisman G, Kolter R, Storz G (1994) The dps promoter is activated by OxyR

during growth and by IHF and sigma S in stationary phase. Mol Microbiol 13: 265-272.

[42] Storz G, Vogel J, Wassarman KM (2011) Regulation by small RNAs in bacteria: expanding frontiers.

Mol Cell 43: 880-891.

[43] Ueguchi C, Misonou N, Mizuno T (2001) Negative control of rpoS expression by

phosphoenolpyruvate: carbohydrate phosphotransferase system in *Escherichia coli*. J Bacteriol 183: 520-

527.

[44] Smith MN, Kwok SC, Hodges RS, Wood JM (2007) Structural and functional analysis of ProQ: an

osmoregulatory protein of *Escherichia coli.* Biochemistry 46: 3084-3095.

[45] Shankar S, Schlictman D, Chakrabarty AM (1995) Regulation of nucleoside diphosphate kinase and

an alternative kinase in *Escherichia coli:* role of the sspA and rnk genes in nucleoside triphosphate

formation. Mol Microbiol 17: 935-943.

[46] Yoshida Y, Sugiyama S, Oyamada T, Yokoyama K, Kim SK, et al. (2011) Identification of PhoB

binding sites of the ybD and ytfK promoter regions in *Escherichia coli*. J Microbiol 49: 285-289.

[47] Feng Y, Cronan JE (2009) *Escherichia coli* unsaturated fatty acid synthesis: complex transcription of

the fabA gene and in vivo identification of the essential reaction catalyzed by FabB. J Biol Chem 284:

29526-29535.

[48] Masters PS, Hong JS (1981) Genetics of the glutamine transport system in *Escherichia coli*. J

Bacteriol 147: 805-819.

[49] Zheng J, Yates SP, Jia Z (2012) Structural and mechanistic insights into the bifunctional enzyme

isocitrate dehydrogenase kinase/phosphatase AceK. Philos Trans R Soc Lond B Biol Sci 367: 2656-2668.

[50] Chi F, Wang Y, Gallaher TK, Wu CH, Jong A, et al. (2009) Identification of IbeR as a stationary-

phase regulator in meningitic Escherichia coli K1 that carries a loss-of-function mutation in rpoS. J

Biomed Biotechnol 2009: 520283.

[51] Mohan S, Kelly TM, Eveland SS, Raetz CR, Anderson MS (1994) An *Escherichia coli* gene (FabZ)

encoding (3R)-hydroxymyristoyl acyl carrier protein dehydrase. Relation to fabA and suppression of

mutations in lipid A biosynthesis. J Biol Chem 269: 32896-32903.

[52] Goldstein J, Pollitt NS, Inouye M (1990) Major cold shock protein of *Escherichia coli.* Proc Natl Acad

Sci U S A 87: 283-287.

[53] Zheng R, Blanchard JS (2000) Kinetic and mechanistic analysis of the *E. coli* panE-encoded

ketopantoate reductase. Biochemistry 39: 3708-3717.

[54] Battesti A, Majdalani N, Gottesman S (2011) The RpoS-mediated general stress response in

*Escherichia coli.* Annu Rev Microbiol 65: 189-213.

[55] Allen GC, Jr., Kornberg A (1993) Assembly of the primosome of DNA replication in *Escherichia coli*. J

Biol Chem 268: 19204-19209.

[56] Ferrieres L, Aslam SN, Cooper RM, Clarke DJ (2007) The yjbEFGH locus in *Escherichia coli* K-12 is

an operon encoding proteins involved in exopolysaccharide production. Microbiology 153: 1070-1080.

[57] Landick R, Yanofsky C, Choo K, Phung L (1990) Replacement of the *Escherichia coli* trp operon

attenuation control codons alters operon expression. J Mol Biol 216: 25-37.

[58] Ma L, Payne SM (2012) AhpC is required for optimal production of enterobactin by *Escherichia coli*. J

Bacteriol 194: 6748-6757.

[59] Weitz D, Harder D, Casagrande F, Fotiadis D, Obrdlik P, et al. (2007) Functional and structural

characterization of a prokaryotic peptide transporter with features similar to mammalian PEPT1. J Biol

Chem 282: 2832-2839.

[60] Benjdia A, Deho G, Rabot S, Berteau O (2007) First evidences for a third sulfatase maturation

system in prokaryotes from E. coli aslB and ydeM deletion mutants. FEBS Lett 581: 1009-1014.

[61] Seputiene V, Motiejunas D, Suziedelis K, Tomenius H, Normark S, et al. (2003) Molecular

characterization of the acid-inducible asr gene of *Escherichia coli* and its role in acid stress response. J

Bacteriol 185: 2475-2484.

[62] Schauer AT, Cheng SW, Zheng C, St Pierre L, Alessi D, et al. (1996) The alpha subunit of RNA

polymerase and transcription antitermination. Mol Microbiol 21: 839-851.

[63] Jenkins LS, Nunn WD (1987) Genetic and molecular characterization of the genes involved in short-

chain fatty acid degradation in *Escherichia coli*: the ato system. J Bacteriol 169: 42-52.

[64] Lund PA (2009) Multiple chaperonins in bacteria--why so many? FEMS Microbiol Rev 33: 785-800.

[65] Kornberg HL (1990) Fructose transport by *Escherichia coli*. Philos Trans R Soc Lond B Biol Sci 326:

505-513.

[66] Yang L, Liao RZ, Yu JG, Liu RZ (2009) DFT study on the mechanism of *Escherichia coli* inorganic

pyrophosphatase. J Phys Chem B 113: 6505-6510.

[67] Carter EL, Jager L, Gardner L, Hall CC, Willis S, et al. (2007) *Escherichia coli* abg genes enable

uptake and cleavage of the folate catabolite p-aminobenzoyl-glutamate. J Bacteriol 189: 3329-3334.

[68] Newman J, Seabrook S, Surjadi R, Williams CC, Lucent D, et al. (2013) Determination of the

structure of the catabolic N-succinylornithine transaminase (AstC) from *Escherichia coli.* PLoS One 8:

e58298.

[69] Cusa E, Obradors N, Baldoma L, Badia J, Aguilar J (1999) Genetic analysis of a chromosomal region

containing genes required for assimilation of allantoin nitrogen and linked glyoxylate metabolism in

*Escherichia coli.* J Bacteriol 181: 7479-7484.

[70] Tan BK, Bogdanov M, Zhao J, Dowhan W, Raetz CR, et al. (2012) Discovery of a cardiolipin

synthase utilizing phosphatidylethanolamine and phosphatidylglycerol as substrates. Proc Natl Acad Sci

U S A 109: 16504-16509.

[71] Dauvillee D, Kinderf IS, Li Z, Kosar-Hashemi B, Samuel MS, et al. (2005) Role of the *Escherichia coli*

glgX gene in glycogen metabolism. J Bacteriol 187: 1465-1473.

[72] Banach-Orlowska M, Fijalkowska IJ, Schaaper RM, Jonczyk P (2005) DNA polymerase II as a fidelity

factor in chromosomal DNA synthesis in *Escherichia coli.* Mol Microbiol 58: 61-70.

[73] Blasco F, Pommier J, Augier V, Chippaux M, Giordano G (1992) Involvement of the narJ or narW

gene product in the formation of active nitrate reductase in *Escherichia coli.* Mol Microbiol 6: 221-230.

[74] Raha M, Kawagishi I, Muller V, Kihara M, Macnab RM (1992) *Escherichia coli* produces a

cytoplasmic alpha-amylase, AmyA. J Bacteriol 174: 6644-6652.

[75] Johnson NA, McKenzie RM, Fletcher HM (2011) The bcp gene in the bcp-recA-vimA-vimE-vimF

operon is important in oxidative stress resistance in Porphyromonas gingivalis W83. Mol Oral Microbiol

26: 62-77.

[76] Szumanski MB, Boyle SM (1992) Influence of cyclic AMP, agmatine, and a novel protein encoded by

a flanking gene on speB (agmatine ureohydrolase) in *Escherichia coli*. J Bacteriol 174: 758-764.

[77] Rath D, Jawali N (2006) Loss of expression of cspC, a cold shock family gene, confers a gain of

fitness in *Escherichia coli* K-12 strains. J Bacteriol 188: 6780-6785.

[78] Matern Y, Barion B, Behrens-Kneip S (2010) PpiD is a player in the network of periplasmic

chaperones in *Escherichia coli.* BMC Microbiol 10: 251.

[79] Zylicz M (1993) The *Escherichia coli* chaperones involved in DNA replication. Philos Trans R Soc

Lond B Biol Sci 339: 271-277; discussion 277-278.

[80] Gronow S, Brabetz W, Brade H (2000) Comparative functional characterization in vitro of

heptosyltransferase I (WaaC) and II (WaaF) from *Escherichia coli*. Eur J Biochem 267: 6602-6611.

[81] Abdel-Hamid AM, Cronan JE (2007) Coordinate expression of the acetyl coenzyme A carboxylase

genes, accB and accC, is necessary for normal regulation of biotin synthesis in *Escherichia coli.* J

Bacteriol 189: 369-376.

[82] Ge W, Wolf A, Feng T, Ho CH, Sekirnik R, et al. (2012) Oxygenase-catalyzed ribosome hydroxylation

occurs in prokaryotes and humans. Nat Chem Biol 8: 960-962.

[83] Sahu SN, Acharya S, Tuminaro H, Patel I, Dudley K, et al. (2003) The bacterial adaptive response

gene, barA, encodes a novel conserved histidine kinase regulatory switch for adaptation and modulation

of metabolism in *Escherichia coli*. Mol Cell Biochem 253: 167-177.

[84] Narita S, Tokuda H (2009) Biochemical characterization of an ABC transporter LptBFGC complex

required for the outer membrane sorting of lipopolysaccharides. FEBS Lett 583: 2160-2164.

[85] Shi IY, Stansbury J, Kuzminov A (2005) A defect in the acetyl coenzyme A<-->acetate pathway

poisons recombinational repair-deficient mutants of *Escherichia coli*. J Bacteriol 187: 1266-1275.

[86] Nenninger AA, Robinson LS, Hammer ND, Epstein EA, Badtke MP, et al. (2011) CsgE is a curli

secretion specificity factor that prevents amyloid fibre aggregation. Mol Microbiol 81: 486-499.

[87] Outten FW, Wood MJ, Munoz FM, Storz G (2003) The SufE protein and the SufBCD complex

enhance SufS cysteine desulfurase activity as part of a sulfur transfer pathway for Fe-S cluster assembly

in *Escherichia coli.* J Biol Chem 278: 45713-45719.

[88] Zhang Y, Shi C, Yu J, Ren J, Sun D (2012) RpoS regulates a novel type of plasmid DNA transfer in

*Escherichia coli.* PLoS One 7: e33514.

[89] Seol W, Shatkin AJ (1991) *Escherichia coli* kgtP encodes an alpha-ketoglutarate transporter. Proc

Natl Acad Sci U S A 88: 3802-3806.

[90] Schneider E, Freundlieb S, Tapio S, Boos W (1992) Molecular characterization of the MalT-

dependent periplasmic alpha-amylase of *Escherichia coli* encoded by malS. J Biol Chem 267: 5148-5154.

[91] Imamura N, Nakayama H (1982) thiK and thiL loci of *Escherichia coli*. J Bacteriol 151: 708-717.

[92] Oldham ML, Chen S, Chen J (2013) Structural basis for substrate specificity in the *Escherichia coli*

maltose transport system. Proc Natl Acad Sci U S A 110: 18132-18137.

[93] Lehmann C, Doseeva V, Pullalarevu S, Krajewski W, Howard A, et al. (2004) YbdK is a carboxylate-

amine ligase with a gamma-glutamyl:Cysteine ligase activity: crystal structure and enzymatic assays.

Proteins 56: 376-383.

[94] Genschik P, Drabikowski K, Filipowicz W (1998) Characterization of the *Escherichia coli* RNA 3'-

terminal phosphate cyclase and its sigma54-regulated operon. J Biol Chem 273: 25516-25526.

[95] Kurihara S, Oda S, Tsuboi Y, Kim HG, Oshida M, et al. (2008) gamma-Glutamylputrescine

synthetase in the putrescine utilization pathway of *Escherichia coli* K-12. J Biol Chem 283: 19981-19990.

[96] Aleshin VV, Zakataeva NP, Livshits VA (1999) A new family of amino-acid-efflux proteins. Trends

Biochem Sci 24: 133-135.

[97] Finkel SE, Kolter R (2001) DNA as a nutrient: novel role for bacterial competence gene homologs. J

Bacteriol 183: 6288-6293.

[98] Klein G, Muller-Loennies S, Lindner B, Kobylak N, Brade H, et al. (2013) Molecular and structural

basis of inner core lipopolysaccharide alterations in *Escherichia coli*: incorporation of glucuronic acid and

phosphoethanolamine in the heptose region. J Biol Chem 288: 8111-8127.

[99] Yamamoto Y, Miwa Y, Miyoshi K, Furuyama J, Ohmori H (1997) The *Escherichia coli* ldcC gene

encodes another lysine decarboxylase, probably a constitutive enzyme. Genes Genet Syst 72: 167-172.

[100] Mnatsakanyan N, Bagramyan K, Trchounian A (2004) Hydrogenase 3 but not hydrogenase 4 is

major in hydrogen gas production by *Escherichia coli* formate hydrogenlyase at acidic pH and in the

presence of external formate. Cell Biochem Biophys 41: 357-366.

[101] Hayashi M, Tabata K, Yagasaki M, Yonetani Y (2010) Effect of multidrug-efflux transporter genes

on dipeptide resistance and overproduction in *Escherichia coli.* FEMS Microbiol Lett 304: 12-19.

[102] Egan SE, Fliege R, Tong S, Shibata A, Wolf RE, Jr., et al. (1992) Molecular characterization of the

Entner-Doudoroff pathway in *Escherichia coli:* sequence analysis and localization of promoters for the

edd-eda operon. J Bacteriol 174: 4638-4646.

[103] Saiki K, Mogi T, Hori H, Tsubaki M, Anraku Y (1993) Identification of the functional domains in heme

O synthase. Site-directed mutagenesis studies on the cyoE gene of the cytochrome bo operon in

*Escherichia coli*. J Biol Chem 268: 26927-26934.

[104] Nagakubo S, Nishino K, Hirata T, Yamaguchi A (2002) The putative response regulator BaeR

stimulates multidrug resistance of *Escherichia coli* via a novel multidrug exporter system, MdtABC. J

Bacteriol 184: 4161-4167.

[105] Bradbury AJ, Gruer MJ, Rudd KE, Guest JR (1996) The second aconitase (AcnB) of *Escherichia*

*coli.* Microbiology 142 ( Pt 2): 389-400.

[106] Chiang SM, Schellhorn HE (2012) Regulators of oxidative stress response genes in *Escherichia coli*

and their functional conservation in bacteria. Arch Biochem Biophys 525: 161-169.

[107] Beigi M, Waltzer S, Fries A, Eggeling L, Sprenger GA, et al. (2013) TCA cycle involved enzymes

SucA and Kgd, as well as MenD: efficient biocatalysts for asymmetric C-C bond formation. Org Lett 15:

452-455.

[108] Kao MC, Di Bernardo S, Nakamaru-Ogiso E, Miyoshi H, Matsuno-Yagi A, et al. (2005)

Characterization of the membrane domain subunit NuoJ (ND6) of the NADH-quinone oxidoreductase

from *Escherichia coli* by chromosomal DNA manipulation. Biochemistry 44: 3562-3571.

[109] Yu BJ, Sung BH, Lee JY, Son SH, Kim MS, et al. (2006) sucAB and sucCD are mutually essential

genes in *Escherichia coli*. FEMS Microbiol Lett 254: 245-250.

[110] Kim HS, Nikaido H (2012) Different functions of MdtB and MdtC subunits in the heterotrimeric efflux

transporter MdtB(2)C complex of *Escherichia coli.* Biochemistry 51: 4188-4197.

[111] Meredith TC, Woodard RW (2005) Identification of GutQ from *Escherichia coli* as a D-arabinose 5-

phosphate isomerase. J Bacteriol 187: 6936-6942.

[112] Zhang HJ, Zhu DD, Li ZL, Sun J, Zhu HL (2011) Synthesis, molecular modeling and biological

evaluation of beta-ketoacyl-acyl carrier protein synthase III (FabH) as novel antibacterial agents. Bioorg

Med Chem 19: 4513-4519.

[113] Sauter M, Bohm R, Bock A (1992) Mutational analysis of the operon (hyc) determining hydrogenase

3 formation in *Escherichia coli*. Mol Microbiol 6: 1523-1532.

[114] De Reuse H, Danchin A (1988) The ptsH, ptsI, and crr genes of the *Escherichia coli*

phosphoenolpyruvate-dependent phosphotransferase system: a complex operon with several modes of

transcription. J Bacteriol 170: 3827-3837.

[115] Salim NN, Feig AL (2010) An upstream Hfq binding site in the fhlA mRNA leader region facilitates

the OxyS-fhlA interaction. PLoS One 5.

[116] Ziervogel BK, Roux B (2013) The binding of antibiotics in OmpF porin. Structure 21: 76-87.

[117] Mann PA, Xiong L, Mankin AS, Chau AS, Mendrick CA, et al. (2001) EmtA, a rRNA

methyltransferase conferring high-level evernimicin resistance. Mol Microbiol 41: 1349-1356.

[118] Sobota JM, Imlay JA (2011) Iron enzyme ribulose-5-phosphate 3-epimerase in *Escherichia coli* is

rapidly damaged by hydrogen peroxide but can be protected by manganese. Proc Natl Acad Sci U S A

108: 5402-5407.

[119] Hondorp ER, Matthews RG (2004) Oxidative stress inactivates cobalamin-independent methionine

synthase (MetE) in *Escherichia coli*. PLoS Biol 2: e336.

[120] Bonomi F, Iametti S, Morleo A, Ta D, Vickery LE (2008) Studies on the mechanism of catalysis of

iron-sulfur cluster transfer from IscU[2Fe2S] by HscA/HscB chaperones. Biochemistry 47: 12795-12801.

[121] Kolb KE, Hein PP, Landick R (2014) Antisense oligonucleotide-stimulated transcriptional pausing

reveals RNA exit channel specificity of RNA polymerase and mechanistic contributions of NusA and

RfaH. J Biol Chem 289: 1151-1163.

[122] Pogliano JA, Beckwith J (1994) SecD and SecF facilitate protein export in *Escherichia coli*. EMBO J

13: 554-561.

[123] Missiakas D, Mayer MP, Lemaire M, Georgopoulos C, Raina S (1997) Modulation of the

*Escherichia coli* sigmaE (RpoE) heat-shock transcription-factor activity by the RseA, RseB and RseC

proteins. Mol Microbiol 24: 355-371.

[124] Lilly AA, Crane JM, Randall LL (2009) Export chaperone SecB uses one surface of interaction for

diverse unfolded polypeptide ligands. Protein Sci 18: 1860-1868.

[125] Babu, Mohan, et al. "Quantitative genome-wide genetic interaction screens reveal global epistatic relationships of protein complexes in *Escherichia coli*." PLoS genetics 10.2 (2014): e1004120.
